# Pilliga Ghosts: The Novel Fungi of the Rivers, Creeks, Lakes, and Dams of the Narrabri Region, Australia

**DOI:** 10.1111/1758-2229.70348

**Published:** 2026-04-28

**Authors:** Kim L. J. Porter, Richard Schinteie, Carla Mariani, Paul Greenfield, Se Gong, Stephen Sestak, Nai Tran‐Dinh, David J. Midgley

**Affiliations:** ^1^ CSIRO Lindfield New South Wales Australia; ^2^ Department of Biological Sciences Macquarie University Sydney New South Wales Australia; ^3^ CSIRO Pullenvale Queensland Australia; ^4^ Centre for Bioinnovation University of the Sunshine Coast Sippy Downs Queensland Australia

**Keywords:** aquatic hyphomycetes, Australia, freshwater fungi, semi‐arid climate, zoosporic

## Abstract

Fungal communities in the freshwater systems of the semi‐arid Narrabri region, Australia, remain largely unexplored despite their crucial role as ecosystem regulators. This study provides the first comprehensive survey of aquatic fungal diversity across the Narrabri region of riverine and lacustrine waters in an area over 2000 km^2^ in size. ITS amplicon sequencing of water samples collected in November 2022 and June 2023 identified 344 OTUs, revealing clear temporal variation, with OTU richness significantly higher in November than in June. Notably, ~73% of sequences could not be reliably assigned to phyla, representing substantial ‘fungal dark matter’, although many of these sequences appear to have affinities with poorly characterised zoosporic fungi. Aquatic hyphomycetes were virtually absent. This observation may be due to regular filtering of conidia by hyporheic or ephemeral flow during the dry season in this region. Notably, this study reported the first Australian record of the ectomycorrhizal fungus, *Laccaria miniata,* detected in a mass fruiting event in June 2023. Furthermore, this study demonstrated that two sponges, previously undocumented in the region, are widespread, alongside a novel gastrotrich species occurring at most locations. Taken together, these findings reveal semi‐arid freshwater systems in Australia are hotspots of unrecognised fungal and eukaryote diversity.

## Graphical abstract

Freshwater systems of the semi‐arid Narrabri region, Australia, are hotspots of novel fungal and eukaryote diversity, dominated by zoosporic fungi and unclassified lineages. ITS‑based DNA and water chemistry analyses across various water bodies revealed strong seasonal patterns, a scarcity of aquatic hyphomycetes that may reflect environmental filtering, and the first local records of sponges (not previously recorded in the area) and novel gastrotrichs; these findings underscore the ecological importance of flood events for shaping microbial communities.

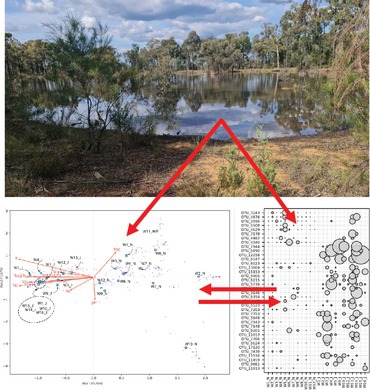

## 1 Introduction

Freshwater fungi have been identified as central regulators of ecosystem health and resilience, yet their diversity and function remain poorly understood. These organisms contribute directly to the self‐purification capacity of aquatic systems, mediating key processes that maintain water quality by metabolising a wide spectrum of xenobiotic compounds (Heilmann‐Clausen et al. 2015; Miglani et al. 2022; Seena et al. 2023). Fungi are also useful for environmental monitoring, as changes in their communities can be early indicators of potential environmental shifts (Barros et al. 2024). Despite these roles and their potential utility, many freshwater fungal species remain poorly described and their responses to changing aquatic environments are poorly documented (Shearer et al. 2007; Bärlocher and Boddy 2016). For fungi, notable aspects of the freshwater habitat are (1) their mainly allochthonous inputs of organic material, (2) their distinct physical niches (i.e., the water column, various epiphytic surfaces, submerged organic materials and sediment), (3) their sometimes ephemeral or hyporheic nature, (4) their flow or stratification, and (5) their lower and more variable oxygen levels (Wetzel 2001).

Aquatic hyphomycetes, also known as Ingoldian fungi after Dr. C. T. Ingold and his pioneering work in the 1940s (Ingold 1942), are arguably the most extensively studied fungi in freshwater systems. These fungi are well known for decomposing submerged plant litter and for their distinctive conidial morphology. Aquatic hyphomycetes typically dominate leaf litter communities (Descals 2020) and are also common in the water column, likely as conidia, with reported spore densities reaching up to 30,000 per litre (Bärlocher and Rosset 1981; Webster and Descals 1981). However, Cornut et al. (2014) demonstrated that hyporheic flow can effectively exclude this group from certain habitats by filtering their spores through the sand bed, which acts as a natural sieve.

Other studies have confirmed the abundance of hyphomycetes in lakes and river waters, but have also identified that these environments host rich zoosporic fungal diversity (Lepère et al. 2019), sometimes more than that of the aquatic hyphomycetes. These zoosporic fungi may have advantages over hyphal forms in the water column as they can use chemotaxis to seek out planktonic material and may then encyst on this floating material, monopolising this resource in a fashion not possible for hyphal fungi (Cooke and Rayner 1984; Laundon and Cunliffe 2021; Powell 1994). A major constraint in our understanding of fungal ecology in freshwater systems is that many species are either poorly studied, known only from a few cultured isolates (Calabon et al. 2022), or belong to novel lineages that are often referred to as ‘fungal dark matter’ (Grossart and Rojas‐Jimenez 2016; Grossart et al. 2016; Sutcliffe et al. 2018; Lepère et al. 2019; Calabon et al. 2022).

In semi‐arid regions, waterbodies take on disproportionately important roles as they represent key hotspots of biodiversity within the landscape (Davis et al. 2013). In Australia, the Narrabri region in northern–central New South Wales encompasses two contrasting landscapes. To the north, fertile vertosols support intensive irrigated cotton and mixed‐cropping systems (Ogle et al. 1993; Rosendahl et al. 2009). To the south and southwest lies the 5000 km^2^ Pilliga Scrub, also known as the Pilliga Woodlands, characterised by nutrient‐poor sands and a mosaic of grazing, aquaculture, conservation reserves, selective logging, mining, and proposed coal‐seam‐gas developments (Paull and Elizabeth 1999; Green et al. 2011). These woodlands are the largest and most continuous semi‐arid woodland in eastern Australia.

Because this region experiences climatic extremes and includes both high‐conservation areas and critical agricultural zones that rely on high‐quality water for productivity, identifying the factors that influence water quality, including microbial influences, is essential. Indeed, the semi‐arid setting of Narrabri makes water the most critical resource, with annual rainfall averaging about 600 mm, mainly during summer, while evaporation exceeds 1400 mm, leaving soils dry for much of the year (Welsh et al. 2014; Bureau of Meteorology 2025). Despite this, the Namoi River and accompanying creeks (Mulgate, Long Gully, Bohena) deliver regulated flows of 0.5–1 GL yr.^−1^ that support both irrigation and ecosystems (Green et al. 2011; NSW Office of Water 2016) while episodic floods such as in 1955, 1971, and October 2022 highlight the region as vulnerable to hydrological extremes. These events, together with drought, influence the ecological and land‐use dynamics of the area (WRM Water and Environment 2016). This study aimed to capture seasonal variation by sampling in November, during the period of typically higher rainfall, and again in June, when conditions are generally drier.

Many of the waterbodies within the Narrabri region are also notably turbid at times. This condition likely arises from several interacting factors, including loosely aggregated sandy soils, erosion and first‐flush events associated with the ephemeral creeks, and the frequent presence of windblown dust across the area. Turbidity is further intensified by extensive disturbance from feral animal activity, particularly pigs, and a widespread network of dirt roads throughout the forest.

Furthermore, in their recent meta‐analysis, Lepère et al. (2019) emphasised the need for additional surveys of aquatic ecosystems to catalogue the remarkable diversity of fungi they harbour. The Narrabri region, where this study was conducted, is renowned for its rich folklore of yowies (Australia's answer to Bigfoot) and ghosts, all enigmatic figures that continue to capture the local imagination. By contrast, the freshwater fungi of the area, though very real and ecologically significant, remain largely unexamined. Much like the elusive creatures of regional legend, these fungi represent mysteries awaiting discovery. This study sought to address this knowledge gap through a systematic survey of riverine and lacustrine sites south and southwest of Narrabri within and around the proposed Narrabri Gas Project area across two timepoints to try and gain an understanding of both biodiversity and temporal changes in aquatic fungal communities in the region. The results are explored in an ecological context.

## 2 Materials and Methods

### 2.1 Study Area

**FIGURE 1:**
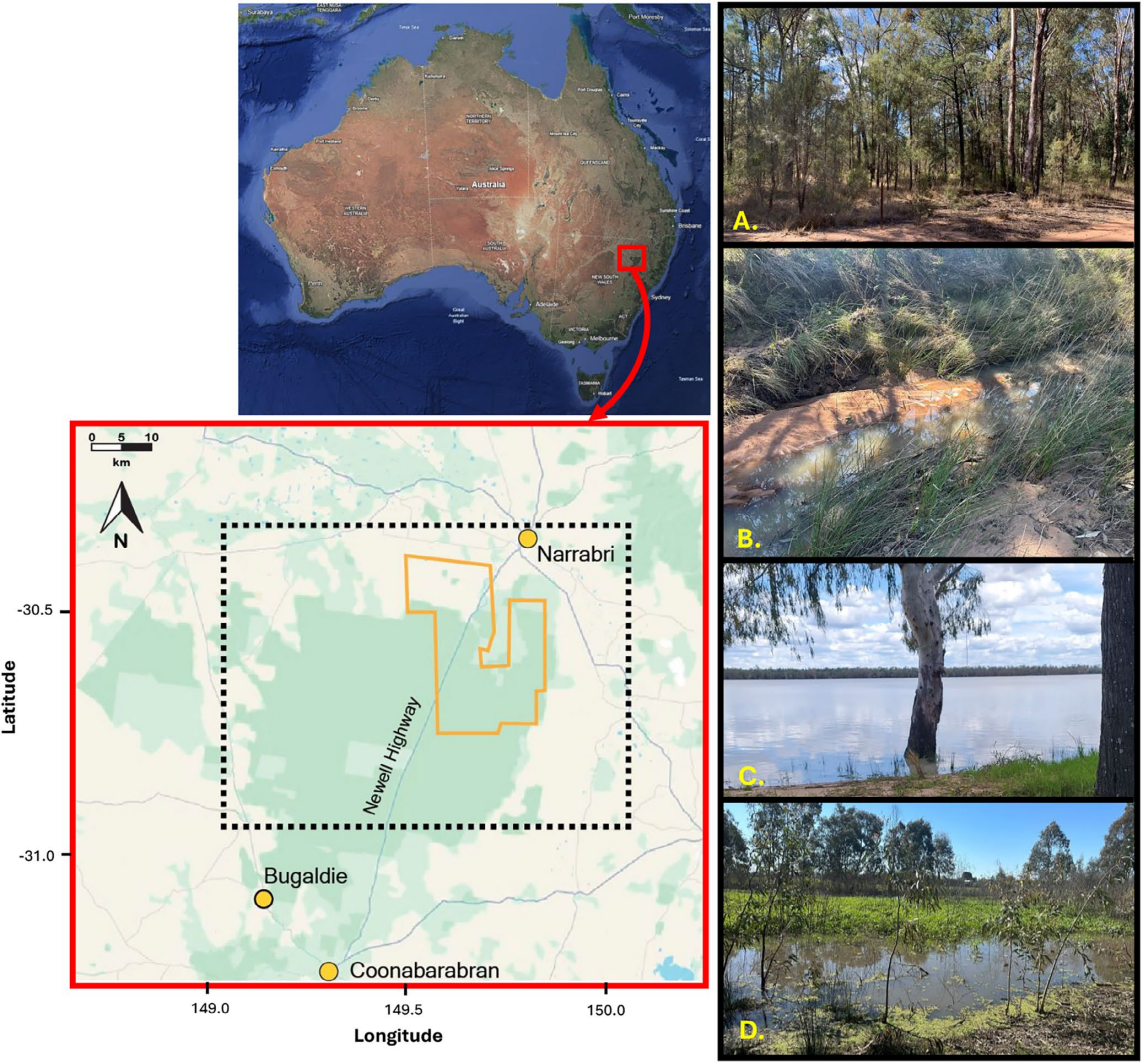
Study site (black dashed inset) and its location in Australia (outset, map of Australia) and the approximate outline of the Narrabri Gas Project area (orange inset) in New South Wales. Map courtesy: Google, 2025. Right top to bottom: (A) typical vegetation of the Pilliga Woodlands, open, dry, sclerophyll forest. (B–D) Freshwater habitats sampled in the present study (B) Tulla Mullen Creek, Narrabri, (C) Yarrie Lake, Narrabri and (D) O′Brien's Creek, Narrabri.

The area of study (Figure 1, black dashed box in inset) is situated around the Narrabri Gas Project (Figure 1, orange outline in inset), a proposed coal seam gas development in northern‐central New South Wales, Australia. Sampling was initially planned within the gas project boundaries, but flooding in November 2022 restricted woodland access, so most collections were made within 200–500 m of major access roads. The township of Narrabri lies north of the project area and occupies the transition between fertile vertosolic soils that support intensive irrigated agriculture, mainly cotton, and sandy, nutrient‐poor soils (Welsh et al. 2014) that sustain a mosaic of grazing, aquaculture, mining, and extensive native woodlands. The fertile vertosolic soils have been the focus of extensive microbiological research (Ogle et al. 1993; McGee et al. 1997; Pattinson and McGee 1997; Mondal et al. 2004; Nehl et al. 2004; Knox et al. 2006, 2008; Loke 2007; Rosendahl et al. 2009; Le et al. 2022). The dominant vegetation of the sandy‐soil woodlands is a mix of *Eucalyptus* species, interspersed with *Casuarina* and native cypress pines (*Callitris* species), with an Acacia midstory and diverse understory plants (Whipp 2009; Benson et al. 2010). The riparian vegetation differs considerably from the broader forest composition, with surveys by Paull (2017) showing an overstory of *Angophora floribunda*, *Callitris columellaris* , and *Eucalyptus blakelyi* with *Acacia deanei* as the major midstory species. Paull (2017) report the riparian understory as being diverse with members of the Asparagaceae (*Lomandra longifolia*), Convolvulaceae ( *Dichondra repens* ), Cyperaceae ( *Gahnia aspera* ), Fabaceae (*Swainsona cadellii*), Malvaceae (*Sida corrugata*), Oxalidaceae (*Oxalis perrenans*), and the Poaceae (*Aristida ramosa*, *Austrostipa setacea*, *Cynodon dactylon* , *Digitaria diffusa*, *Microlaena stipoides* ) present.

### 2.2 Selection of Sampling Sites

**FIGURE 2:**
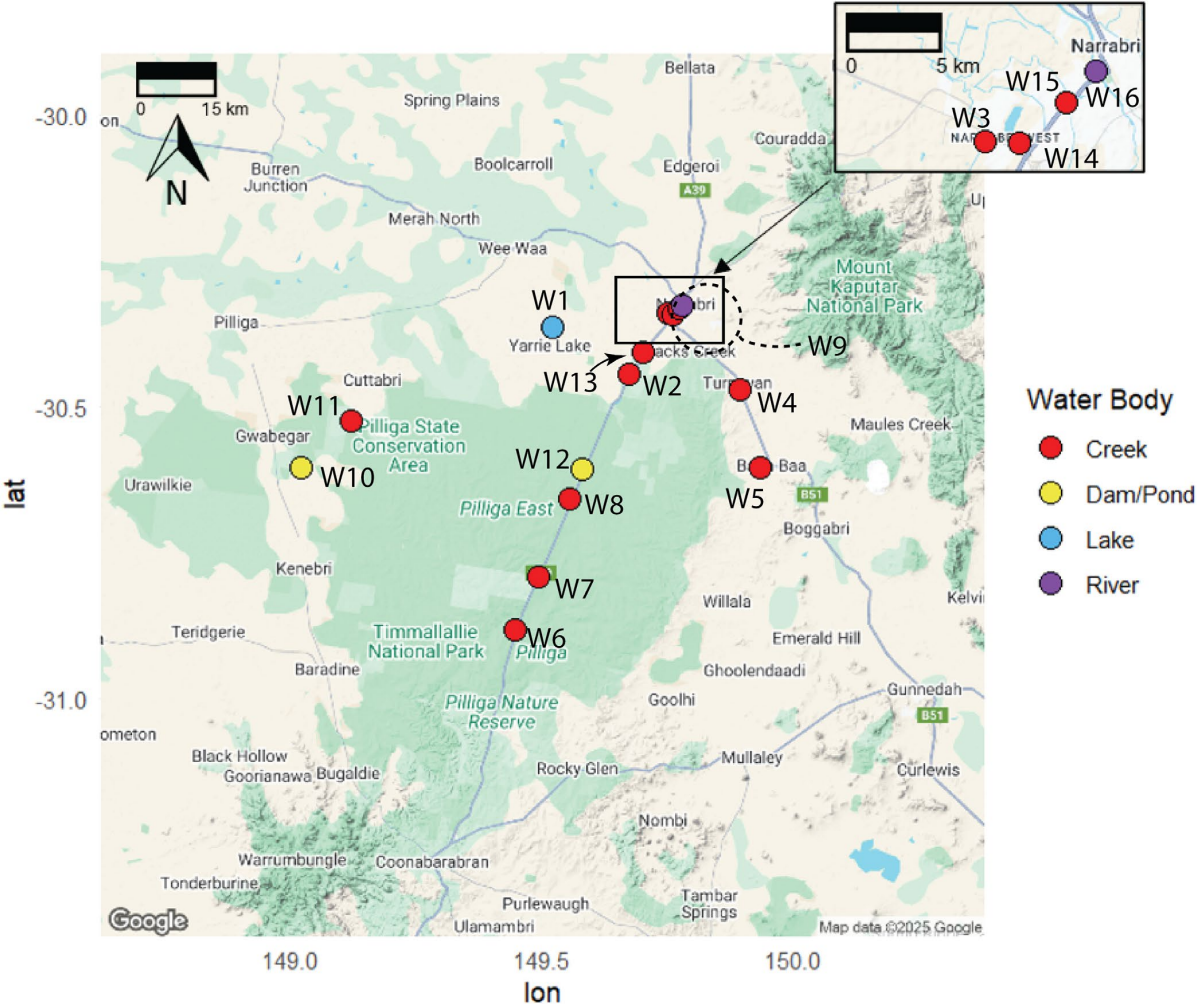
Map showing sample locations and waterbody types (creeks (red), rivers (purple), lakes (blue) along with dams and ponds (yellow)). Note that W16 Narrabri Creek is shown as a ‘river’ as it carries most of the water from the Namoi as it passes the township of Narrabri, while the Namoi River in this area is shown as a ‘creek’. Map courtesy: Google, 2025. Dotted line for W9 indicates its approximate location.

A total of 24 water samples were collected across 16 sites in November 2022 and June 2023 (Figures 1 and 2; Tables 1 and S1). However, not all sites were able to be sampled in both 2022 and 2023, due to access restrictions or the absence of surface water during the 2023 campaign. Eight sites: Yarrie Lake (W1), Bohena Creek (W2), Long Gully (W3), Pine Creek (W4), Tulla Mullen Creek (W5), a private farm dam (W9), Etoo Creek Dam (W10), and X‐Line Dam (W12) were successfully sampled at both timepoints (Figure 2; Table 1). In contrast, Andy's Creek (W6), Wiggans Creek (W7), Duck Creek (W8), and Tinegie Creek (W11) were sampled only in November 2022, as these creeks were not flowing in June 2023. Conversely, Spring Creek (W13), O'Briens Creek (W14), the Namoi River (W15), and Narrabri Creek (W16) were only sampled in June 2023.

**TABLE 1:** Summary of surface water samples collected in the study, including their site locations and descriptive characteristics.

Identifier	Location	Description	Notes
W1	Yarrie Lake (−30.365735, 149.521898)	Lake water, ~ 1 m offshore	Collected in Nov 2022 and June 2023
W2	Bohena Creek (−30.4445927, 149.6731139)	Creek water sample	Collected in Nov 2022 and June 2023
W3	Long Gully (−30.34191102, 149.7519827)	Creek water sample	Collected in Nov 2022 and June 2023
W4	Pine Creek (−30.4707856, 149.8920744)	Creek water sample	Collected in Nov 2022 and June 2023
W5	Tulla Mullen Creek (−30.6048641, 149.9333439)	Creek water sample	Collected in Nov 2022 and June 2023
W6	Andy's Creek (−30.8818921, 149.4452951)	Creek water sample	Collected only during November 2022, due to an absence of water in June 2023.
W7	Wiggans Creek (−30.7911373, 149.4916695)	Creek water sample	Collected only during November 2022, due to an absence of water in June 2023.
W8	Duck Creek (−30.657751, 149.5564804)	Creek water sample	Collected only during November 2022, due to an absence of water in June 2023.
W9	Farm Dam (private land, GPS withheld)	Private farmland, dam water sample	Collected in Nov 2022 and June 2023
W10	Etoo Creek Dam (−30.6068004, 149.0199846)	Dam water sample	Collected in Nov 2022 and June 2023
W11	Tinegie Creek (−30.5242247, 149.1219595)	Creek water sample	Collected only during November 2022, due to an absence of water in June 2023.
W12	Roadside dam/large pond X‐Line Road Dam (−30.6091106, 149.5791971)	Pond samples, large semi‐permanent pond, south of Crow Rd. of the eastern side of the Newell Highway.	Collected in Nov 2022 and June 2023
W13	Spring Creek (−30.407251, 149.700228)	Creek water sample	Only collected in June 2023.
W14	O'Briens Creek (−30.342155, 149.760578)	Creek water sample	Only collected in June 2023.
W15	Namoi River (−30.333446, 149.772475)	Creek water sample	Only collected in June 2023
W16	Narrabri Creek (−30.326504, 149.779678)	River water sample	Only collected in June 2023.

Particular attention should be given to W15 and W16. Although both samples were collected near the township of Narrabri, the hydrological setting is unusual. Northwest of Narrabri, the Namoi River bifurcates into two channels: the Namoi River and Narrabri Creek. Counterintuitively, it is Narrabri Creek, rather than the Namoi River, that carries the majority of the flow until the two channels rejoin southeast of the township. According to local knowledge, this reversal of flow dominance may be linked to human modifications made at the branch point during or before the 1950s. While no data was found to independently verify this claim, the incongruous naming of the ‘creek’ and the ‘river’, combined with local accounts, suggests that historically the Namoi River likely conveyed the main watercourse.

### 2.3 Sampling Procedure

To ensure consistency and prevent contamination, all water sampling was undertaken aseptically. Prior to collection, sampling equipment was cleaned and decontaminated using ethanol, and fresh nitrile or latex gloves (both powder free) were used for handling samples. All samples were collected from the water column with care to avoid including any visible debris, especially plant material. For microbial profiling, all surface water samples were collected in 500 mL Schott bottles (with Teflon seals in lids), containing a preservation medium composed of dimethyl sulfoxide, disodium ethylenediaminetetraacetic acid (EDTA), and saturated salts (DESS) (Seutin et al. 1991; Yoder et al. 2006). DESS has been demonstrated to adequately prevent significant changes in community structure post collection (Rachel and Gieg 2020). In brief, a 500 mL autoclaved Schott bottle was prepared containing 100 mL of dimethyl sulfoxide, 46.5 g of EDTA, and 50 g of NaCl, after which Grade 1 Milli‐Q water was added to bring the total volume to 200 mL. The pH was adjusted to approximately 8 by adding 14 mL of a 10 M NaOH solution. During sampling, ~300 mL of collected water was introduced to each bottle containing the DESS reagents, bringing the final volume to 500 mL. For water chemistry, all samples were placed into dedicated collection bottles supplied by Australian Laboratory Services (ALS Environmental; https://www.alsglobal.com/en/australia) following the service provider's directions for collection. Water chemistry analyses were conducted on all samples including pH, electrical conductivity, alkalinity, major cations and anions, total organic carbon, total nitrogen, total phosphorus, mercury, fluoride, ammonia, bromide, and total metals.

### 2.4 DNA Extraction, Amplification, and Sequencing

#### 2.4.1 DNA Extraction

Collected DESS‐preserved surface water samples were passed through Millipore 0.1 μm polyvinylidene (PVDF) filter membranes (Merck, Darmstadt, Germany) to concentrate microbial cells. From each membrane, a 5 mm strip was cut, finely sliced, and placed into the bead‐beating tubes provided with the DNA extraction kit. DNA was subsequently extracted from these sliced membranes using the DNeasy PowerLyzer PowerSoil Kit (Qiagen, VIC, Australia) according to the standard protocol with only minor adjustments. This modified procedure included an additional 10‐min incubation at 70°C while shaking in an Eppendorf Thermomixer before bead beating (FastPrep‐24 5G, MP Biomedicals).

#### 2.4.2 Polymerase Chain Reaction (PCR) and Amplicon Sequencing

Fungal community composition was assessed by amplifying the Internal Transcribed Spacer (ITS) region with primers ITS1‐F (5′‐CTTGGTCATTTAGAGGAAGTAA‐3′) (Gardes and Bruns 1993) and ITS2 (5′‐GCTGCGTTCTTCATCGATGC‐3′) (White et al. 1990). Resulting amplicons were sequenced on an Illumina MiSeq using 2 × 250 bp paired‐end chemistry at Molecular Research LP Laboratories (Shallowater, Texas).

#### 2.4.3 Bioinformatics

Sequences were subsequently processed using the Greenfield Hybrid Amplicon Pipeline (GHAP; Greenfield 2017). Analyses for ITS followed a standard workflow. In brief, data were demultiplexed, subjected to quality control prior to merging, clustered into Operational Taxonomic Units (OTUs) using USEARCH v11 (USEARCH; Edgar 2010) with a > 97% identity radius. Reads were then mapped back to the resultant OTUs to form an OTU table. Classification of representative OTU sequences was undertaken using the Warcup and UNITE datasets (Deshpande et al. 2016; Kõljalg et al. 2020; Abarenkov et al. 2024) for the RDP Classifier (Wang et al. 2007), along with matching to a manually curated version of the NCBI RefSeq fungal database (O'Leary et al. 2015). For this study, a combined analysis of soil and water samples was used to delineate environment‐specific OTUs, and only those found exclusively in water were included to reduce artefacts caused by the transfer of soil fungi during floods.

### 2.5 Determination of Water‐Only OTUs

The study collected DNA from both soil and water samples, and these were prepared and sequenced together in a single run. Resulting sequence data were processed through the GHAP amplicon pipeline three times: once for the combined set of samples (including soil and water), once for the water samples alone, and once for the soil samples alone (Table 2). These three independent runs provided better control over possible cross‐contamination between soil and water samples and helped identify soil fungi that may have been transported into the water during the flood events of November 2022.

**TABLE 2:** Example soil/water OTU tagging.

Combined Soil + Water OTU	Soil OTU	% Reads in soil	Water OTU	% Reads in water	Mapping	
					Soil	Water
OTU_2 (# reads: 704963)	OTU_2 (# reads: 703060)	99.73%	OTU_148 (# reads: 1898)	0.27%	Soil‐only	
OTU_59 (# reads: 40419)	OTU_2423 (# reads: 269)	0.67%	OTU_2 (# reads: 40162)	99.36%		Water‐only
OTU_48 (# reads: 50111)	OTU_926 (# reads: 1748)	3.49%	OTU_3 (# reads: 48364)	96.51%	Soil and water OTU	Soil and water OTU

As this study focused on aquatic fungi, only OTUs identified as restricted to water environments were selected for further analysis. The work was conducted alongside a complementary investigation of fungi obtained from soil in the same region. Using the complete dataset from both soil and water samples, a combined analysis was performed to delineate environment‐specific OTUs, after which only those exclusive to water were retained to reduce potential artefacts caused by flood‐related transfer of soil fungi.

### 2.6 Statistical Analysis

All statistical analyses and data visualisation were performed using RStudio (Posit Team 2025), PAST3 (Hammer et al. 2001), and Python 3.x (ver. 3.9.21; Van Rossum and Drake 2009). In R, data exploration and normality tests were undertaken using base R packages (R Core Team 2017). Permutational Multivariate Analysis of Variance (PERMANOVA) was used to test for differences in microbial community composition among timepoints, using the OTU abundance matrix as the response variable and sampling timepoint (June versus November) as the explanatory variable. Similarity Percentages (SIMPER) analysis identified the OTUs that contributed most to the observed differences between timepoints, based on Bray–Curtis dissimilarities. Multivariate dispersion (PERMDISP) was used to evaluate variation within groups across timepoints. Univariate comparisons (*t*‐tests) were applied to diversity indices to test differences between timepoints, using diversity metrics as response variables and timepoint as the explanatory variable. SIMPER, PERMDISP, *t*‐tests, and diversity metric analyses were conducted in the vegan package (Oksanen et al. 2022). The Bray–Curtis dissimilarity index was applied for all analyses. Figures were prepared from ordination data generated in PAST3 for non‐metric multidimensional scaling (NMDS) and in R for Canonical Correspondence Analysis (CCA). These ordination outputs, together with data for the bubble plot, were visualised as scatter or bubble plots in Python 3.x using Matplotlib (ver. 3.9.2; Hunter 2007) and Pandas (ver. 2.2.3; The Pandas Development Team 2020). Analyses that included OTUs were conducted using normalised relative abundance data obtained from read counts throughout the study.

### 2.7 Accessioning and Data Availability

Data from the present study were deposited in the GenBank database under BioProject PRJNA1269639. All accession numbers are available in Table S2. The complete dataset is available through the CSIRO Data Access Portal (https://data.csiro.au/collection/csiro:65792).

## 3 Results

### 3.1 Water Chemistry at Sampling Sites

Almost all the water samples examined contained very few dissolved salts. Indeed, the highest electrical conductivities (EC) recorded were ~500 μS cm^−1^ in samples collected from Narrabri Creek and the Namoi River near Narrabri. Due to extensive flooding in November 2022, all samples taken at that time contained lower dissolved salt concentrations compared to June 2023 (Table 3). For example, the EC at site W2 was 266 μS cm^−1^ in November 2022, increasing by approximately 30% to 386 μS cm^−1^ in June 2023. Similar patterns were evident for all samples that were collected at both timepoints.

**TABLE 3:** Abridged chemistry of the surface waters in this study. All values are mg L^−1^, unless otherwise specified.

Identifier	pH^a^	EC^b^	HCO_3_^−^	SO_4_^2−^	Cl^−^	Ca^2+^	Mg^2+^	Na^+^	K^+^	Fe	Total N	Total P	Total OC^c^	Total IC^d^
W1_N	5.3	109	26	0	6	4	2	11	6	16.90	1.6	0.29	34	2
W1_J	7.1	169	70	3	10	5	3	18	8	4.65	1.9	0.47	5	21
W2_N	7.3	266	54	0	41	11	11	22	4	5.90	1.0	0.16	19	12
W2_J	7.1	386	104	0	56	15	16	29	4	3.24	1.6	0.10	26	26
W3_N	6.5	96	26	0	9	2	3	13	3	21.30	2.2	0.45	16	8
W3_J	6.7	176	49	2	21	3	2	26	4	45.40	6.1	0.71	18	20
W4_N	7.4	161	68	0	11	9	4	16	5	3.03	1.0	0.13	15	14
W4_J	7.2	289	123	8	14	20	7	23	6	0.29	0.6	0.04	8	30
W5_N	7.3	181	51	0	23	6	4	22	5	11.60	1.6	0.22	25	11
W5_J	6.6	178	43	8	23	3	2	21	7	37.20	7.1	0.83	22	19
W6_N	6.9	195	36	0	34	1	3	30	1	9.10	1.9	0.12	38	9
W7_N	6.9	143	25	0	25	0	2	26	2	6.48	1.4	0.08	35	4
W8_N	7.1	231	37	0	42	5	6	33	2	7.63	2.5	0.12	54	8
W9_N	6.6	44	11	0	6	0	0	5	2	1.60	1.0	0.08	12	2
W9_J	6.4	78	13	1	12	0	0	9	3	3.13	3.1	0.17	27	3
W10_N	7.4	204	57	0	28	1	2	37	1	6.04	1.4	0.12	12	13
W10_J	7.1	307	106	0	32	5	3	48	9	13.20	7.4	0.77	60	24
W11_N	6.5	172	31	0	30	5	3	23	5	8.47	1.8	0.20	44	6
W12_N	6.2	38	7	0	5	0	1	3	2	2.21	1.2	0.07	14	2
W12_J	6.4	73	12	0	13	0	2	8	3	0.12	1.3	0.05	29	2
W13_J	6.4	122	21	5	22	0	0	20	1	9.02	0.0	0.58	6	9
W14_J	6.8	186	66	7	14	5	3	23	5	41.70	9.5	1.90	19	16
W15_J	7.8	681	259	0	61	43	23	57	7	0.70	6.2	0.07	14	69
W16_J	7.2	599	166	46	53	40	22	42	2	1.37	1.2	0.09	44	30
Mean (±SE)	6.84 (±0.11)	211.83 (±31.89)	60.88 (±11.76)	3.33 (±1.93)	24.62 (±3.33)	7.62 (±2.36)	5.17 (±1.31)	23.54 (±2.70)	4.04 (±0.47)	10.85 (±2.65)	2.69 (±0.52)	0.33 (±0.08)	24.83 (±3.04)	15.00 (±2.97)

Abbreviation: SE, Standard error.

^a^ pH units.

^b^ Electrical conductivity in μS cm^−1^; combined Fe^2+^ and Fe^3+^ oxidation states.

^c^ Organic carbon.

^d^ Inorganic carbon.

Most samples were mildly acidic to neutral, with pH values ranging from 6.17 to 7.45. An exception was the November 2022 sample from Yarrie Lake (W1), which had a lower pH of ~5.3. This tea‐coloured sample presumably contained significant tannic acid, absent in later collections, and its relatively high organic carbon content supports this supposition (Table 3).

Major cations were predominantly sodium and potassium, although some samples (W2, W4, and W15) showed a more balanced mixture of calcium/magnesium and sodium/potassium. Sulphate concentrations were generally low, with bicarbonate or bicarbonate mixed with chloride dominating the anions.

Median total nitrogen and phosphorus concentrations of 1.6 and 0.14 mg L^−1^, respectively, were recorded. However, several sites in June 2023 (W3, W5, and W14) showed elevated levels of these elements, with total nitrogen reaching up to 9.5 mg L^−1^ and total phosphorus up to 1.9 mg L^−1^. These sites also exhibited increased transition metals, including lead, particularly in 2023 (Tables 3 and S3), although repeat testing in 2024 indicated a decline in these concentrations (Schinteie et al. 2024).

When comparing all samples collected in November 2022 and June 2023, only EC and total nitrogen showed significant differences (*t*‐test; both *p* < 0.03). EC and total nitrogen were higher in the June collections (EC mean 86 mg L^−1^ compared with 35 mg L^−1^; total nitrogen mean 3.8 mg L^−1^ compared with 1.6 mg L^−1^), while other analytes did not differ significantly.

### 3.2 Identity and Seasonal Dynamics of Eukaryotic OTUs

All samples yielded significant quantities of amplified DNA and were successfully sequenced using 2 × 250 bp paired‐end MiSeq Illumina technology. OTU clustering and taxonomic mapping revealed many novel sequences that did not match any existing entries in GenBank or other databases (Table S2). In total, 2,588,996 read pairs were recovered from water samples, and after quality control, 344 high‐quality OTU sequences were obtained from water‐only samples that were analysed from the sampling sites in this study. Among the 344 OTUs, 264 were detected in the November 2022 collections, while 159 were recorded in the June 2023 collection. The complete dataset, including OTUs excluded by GenBank, is available through the CSIRO Data Access Portal (https://data.csiro.au/collection/csiro:65792).

**FIGURE 3:**
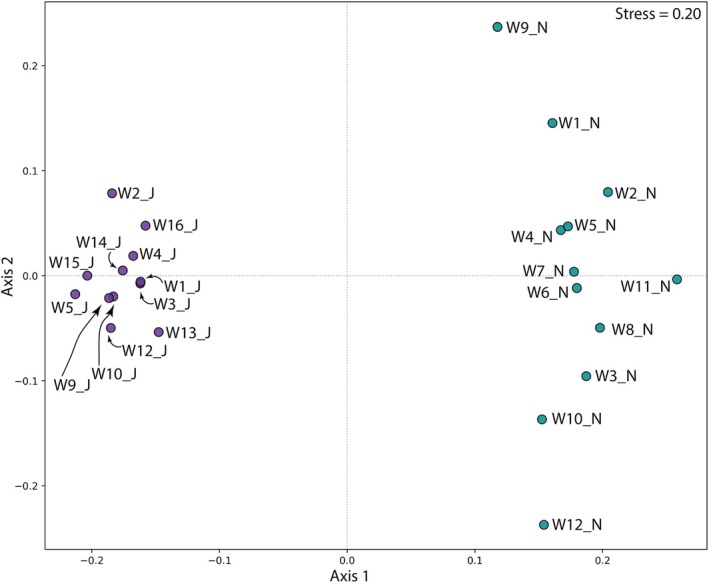
NMDS ordination of communities from the samples obtained in the present study. Coloured groups show significant differences as determined by PERMANOVA (*p* < 0.05).

OTU richness was significantly higher in November 2022, with an average of 159 ± 6 OTUs per sample, compared to just 49 ± 4 OTUs in June 2023 (*t*‐test *p* < 0.0001; Tables 4 and 5). Within each sampling period, richness varied among sites, ranging from 124 OTUs at sites W6_N and W9_N to 180 OTUs at W4_N in November, and from 25 OTUs at W1_J to 82 OTUs at W13_J in June. Despite these differences, overall diversity remained broadly similar between the two sampling periods, while evenness was significantly higher in the June samples. Significant shifts in taxonomic composition were observed over time (PERMANOVA *p* < 0.0001). Multivariate dispersion varied significantly among timepoints (PERMDISP, *F* = 61.54, *p* < 0.001), with November samples showing greater within‐group variability, indicated by a higher mean distance to the group centroid (0.667) compared with June (0.562) (Figure 3).

**TABLE 4:** Taxonomic summary of OTUs recovered across all samples, grouped by major fungal phyla, minor fungal phyla, and non‐fungal eukaryotes.

Organism	All	Nov‐22	Jun‐23
Fungi—Major Phyla			
Ascomycota	87	68	30
Basidiomycota	20	14	8
Chytridiomycota	65	49	33
Cryptomycota	32	24	12
Fungi—Minor Phyla			
Aphelidiomycota	1	0	1
Blastocladiomycota	2	2	0
Entorrhizomycota	1	1	0
Mucoromycota	3	2	1
Zoopagomycota	1	1	0
Unknown fungi	44	29	22
Non‐Fungal Eukaryotes			
Gastrotricha	1	1	1
Porifera	2	2	1
Unknown Eukaryota sp.	85	71	50

**TABLE 5:** Species richness, diversity and evenness per sample.

Sample	Richness^a^	Diversity^b^	Evenness^c^
W1_N	164	0.79	0.16
W1_J	25	0.80	0.25
W2_N	173	0.52	0.10
W2_J	48	0.88	0.23
W3_N	175	0.71	0.14
W3_J	53	0.89	0.22
W4_N	180	0.85	0.16
W4_J	49	0.76	0.19
W5_N	164	0.96	0.19
W5_J	49	0.61	0.16
W6_N	124	0.96	0.20
W7_N	140	0.90	0.18
W8_N	172	0.83	0.16
W9_N	124	0.35	0.07
W9_J	46	0.89	0.23
W10_N	180	0.83	0.16
W10_J	36	0.77	0.22
W11_N	159	0.74	0.15
W12_N	156	0.65	0.13
W12_J	47	0.85	0.22
W13_J	82	0.92	0.21
W14_J	64	0.92	0.22
W15_J	32	0.90	0.26
W16_J	55	0.95	0.24
Mean (±SE) Nov	159.2 ± 5.75	0.76 ± 0.05	0.15 ± 0.01
Mean (±SE) Jun	48.8 ± 4.28	0.84 ± 0.75	0.22 ± 0.007

Abbreviation: SE, Standard error.

^a^ Richness values are the number of OTUs in a sample.

^b^ Diversity values are reported as Simpsons index (1‐D).

^c^ Evenness values are calculated using Simpsons index.

**FIGURE 4:**
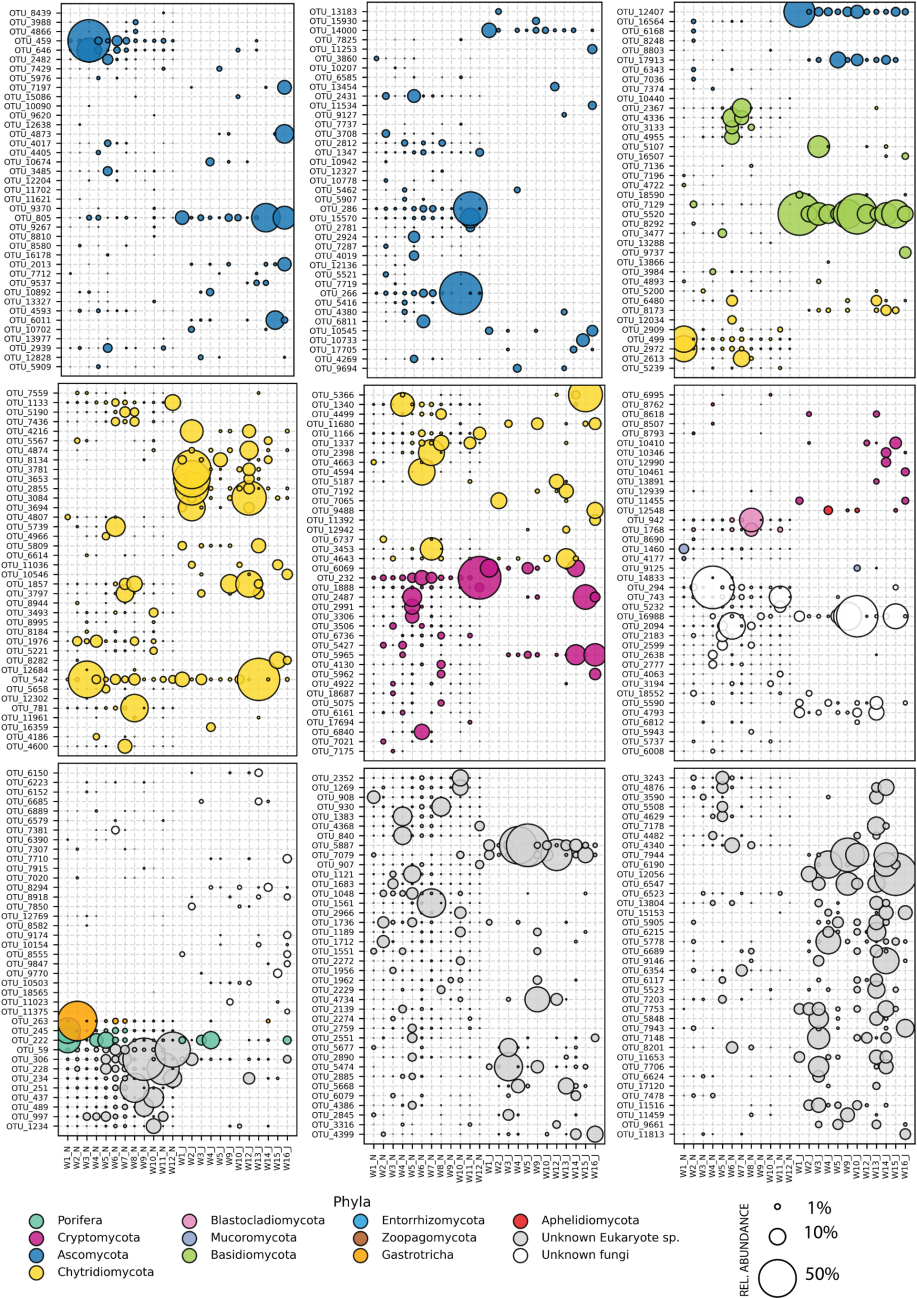
Bubble plot of the OTUs present in the water samples collected during November 2022 and June 2023, coloured and sorted by phylum. The diameter of the bubble indicates the relative abundance in a given sample. N = November sample (e.g., W2_N); J = June sample (e.g., W2_J).

Across both sampling periods, the largest proportion of sequences belonged to unclassified fungi (44 OTUs) and unclassified eukaryotes (85 OTUs), followed by Ascomycota (87 OTUs; 25.3%), Chytridiomycota (65 OTUs; 18.9%), Rozellomycota (32 OTUs; 9.3%), and Basidiomycota (20 OTUs; 5.8%) (Table 4 and Figure 4). Many unclassified fungal and eukaryotic OTUs appear to show weak affinities to various zoosporic lineages, including chytrids, rozellids, microsporids, and aphelids, although this group of OTUs may also contain non‐fungal lineages such as Ichthyosporea (Table S4 and Figure 4). All major phyla exhibited declines in OTU counts between November and June, with Ascomycota decreasing from 68 to 30 OTUs and Chytridiomycota from 49 to 33 (Table 4 and Figure 4). Minor fungal phyla, typically represented by 1–3 OTUs each, were present in low abundance at both time points but were more common in the November samples. Notable, a single Aphelidiomycota OTU appeared exclusively in June.

SIMPER analysis identified the OTUs contributing most to the differences between the November and June collections (Table 6). Twenty OTUs accounted for around half of the total dissimilarity. The most prominent OTU was a strain of *Laccaria minuta* (OTU_5520), a terrestrial ectomycorrhizal fungus absent in November but present in June at an average abundance of 13.52% (Figure 4). Another 19 OTUs (5887, 306, 263, 59, 7079, 16,988, 222, 228, 459, 542, 805, 251, 266, 294, 12,407, 5474, 245, 1561 and 4734) were also key contributors to dissimilarity. Most taxa show clear variation between June and November (Table S5). However, two taxa, OTU_222 (a sponge species) and OTU_542 (a Lobulomycetales chytrid), display high variability within groups. Although these taxa contribute to the overall mathematical dissimilarity, they may not represent ecologically meaningful drivers of difference (Warton et al. 2012).

**TABLE 6:** Major OTUs contributing to compositional dissimilarities between November 2022 and June 2023 collections.

OTU ID	Closest taxonomic match	Taxonomic group	Key characteristics/ecological note	Relative contribution (%)
OTU_5520	*Laccaria minuta*	Basidiomycota	Terrestrial ectomycorrhizal species; dominant in June	6.76
OTU_5887	Unclassified fungus	Unknown (likely zoosporic)	Novel sequence, unassigned phylum	5.27
OTU_306	Unclassified fungus	Unknown	Highly abundant in November 2022	4.05
OTU_263	*Chaetonotus* sp. (Gastrotrich)	Animalia	Likely misidentified algal sequence; benthic grazer	3.01
OTU_59	Unclassified fungus	Unknown	Novel, possibly zoosporic	2.90
OTU_7079	Unclassified fungus	Unknown	Appeared across both periods	2.82
OTU_16988	Unclassified fungus	Unknown	Novel sequence, low similarity to known taxa	2.69
OTU_222	*Heterorotula multidentata*	Porifera	Freshwater sponge (Lake Biwa lineage)	2.51
OTU_228	Unclassified fungus	Unknown	Likely zoosporic	2.34
OTU_459	Unclassified fungus	Unknown	Novel sequence	2.30
OTU_542	Chytrid (Lobulomycetales)	Chytridiomycota	Present in 23 of 24 samples	1.94

Other abundant, albeit poorly resolved, OTUs observed in November included fungal sequences OTU_251, OTU_266, and OTU_294. OTU_251 is related to *Quaeritorhiza* species but distinct (90% ITS identity to NR_171267), OTU_266 likely belongs to the Pleosporaceae family (100% identity to an Arabidopsis endophyte; Dovana et al. 2015), and OTU_294 is an undescribed chytrid closely related to a pond‐derived sequence from Sydney, Australia (GenBank sequence: MF965401; Sutcliffe et al. 2018). Among the top 10 OTUs in June, alongside previously discussed taxa, were OTU_805, identified as a species of *Didymella* possibly *D. keratinophyla*, OTU_3781, a basidiomycete yeast of the genus *Teunia*, OTU_4734, a chytrid related to isolate JEL0341 (GenBank: MT730699) baited with pollen, OTU_5474, an unclassified eukaryotic ITS sequence without close matches, and OTU_12407, closely related to *Saccharomyces cerevisiae* ‘CBS 1171’ but likely representing a different species.

Among all 344 observed OTUs, only one clearly affiliated with traditional aquatic hyphomycetes: OTU_4873, identified as *Campylospora chaetocladia*, known for its ability to degrade submerged leaves. This OTU was found in significant abundance exclusively in Narrabri Creek, one of the two anabranches of the Namoi River around the township of Narrabri (and the one that carries the majority of the flow) around the township of Narrabri.

Several non‐fungal OTUs were also identified in the collections. OTU_222 showed a 100% ITS sequence match to *Heterorotula multidentata* (GenBank EF151933; EF151941). OTU_245 matched *Anheteromeyenia argyrosperma* (GenBank PV788123) with 99.68% ITS identity and appeared in all November 2022 samples, reaching its highest abundance at Yarrie Lake (W1). OTU_263 produced top BLAST matches to *Boldia erythrosiphon* (AF087109; AF087113) and additional hits to gastrotrichs within *Chaetonotus*, with the closest described gastrotrich match being *Chaetonotus gulosus* (OM421697) at 91% ITS identity.

**FIGURE 5:**
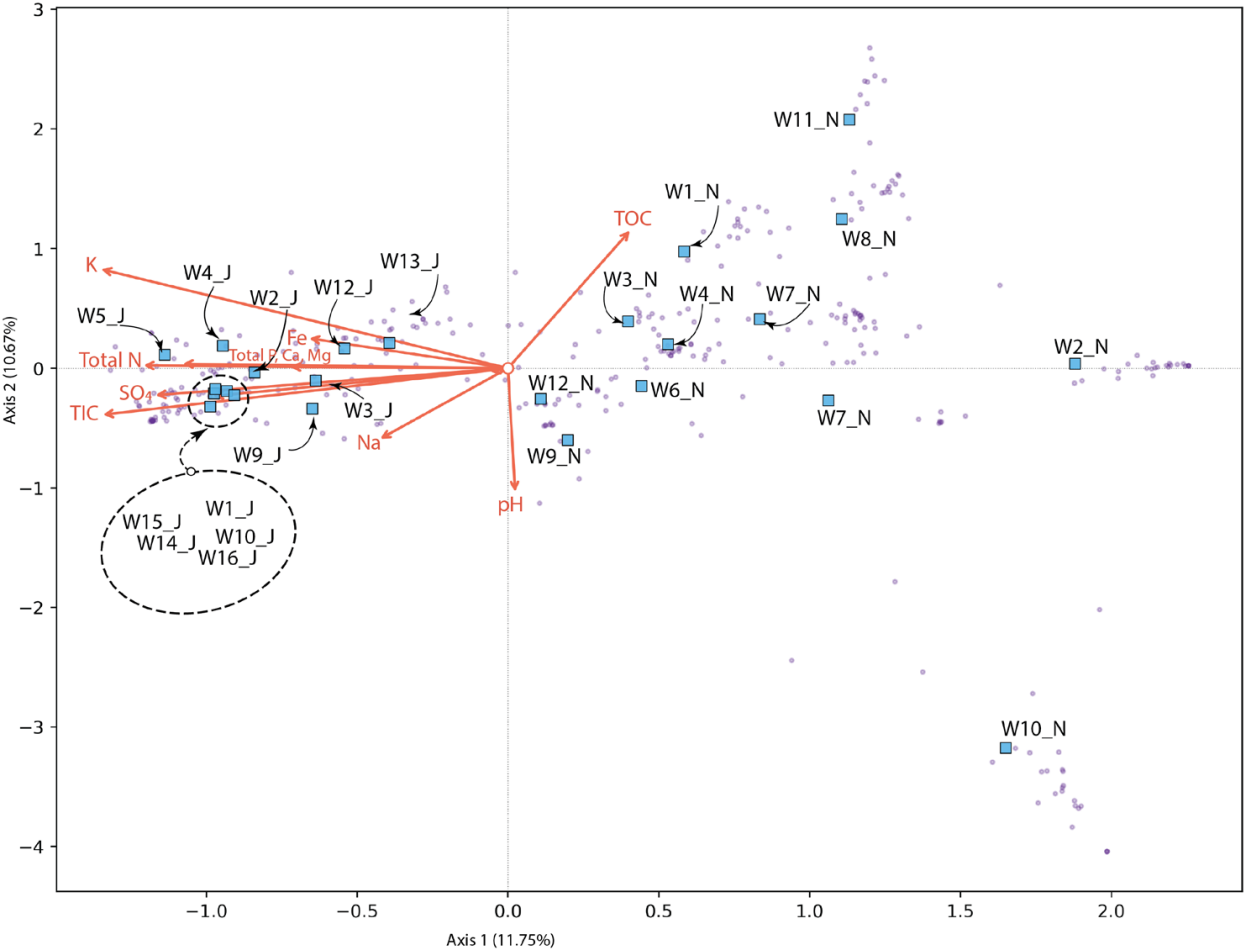
Canonical correspondence analysis (CCA) showing sampling sites (blue squares) and OTUs (purple dots) with reference to water chemistry.

Chemically, samples collected in November 2022 showed generally lower solute concentrations than those collected in June 2023 (Figure 5). Canonical Correspondence Analysis (CCA) revealed patterns consistent with non‐metric multidimensional scaling (NMDS), showing a primary distinction between the two sampling periods (Figure 3). This observation suggests that water chemistry affects microbial community composition and exerts strong environmental filtering on OTU assemblages.

## 4 Discussion

Data from the present study demonstrate for the first time that riverine and lacustrine waters of the Narrabri region host diverse and poorly characterised microbiomes. Indeed, among the 344 OTUs detected in this study, the largest group was comprised of ‘unclassified’ fungi, followed by zoosporic fungi, and then Ascomycota.

The observation of numerous ‘unclassified fungi’ aligns with studies from various regions around the world that have reported a similar abundance of ‘fungal dark matter’ (Grossart and Rojas‐Jimenez 2016; Grossart et al. 2016; Sutcliffe et al. 2018; Lepère et al. 2019). Analyses in this study indicate that some of these unclassified lineages likely included probable chytrid, rozellid, and aphelid sequences, along with a variety of other organisms, including some that were clearly non‐fungal. Previous studies have suggested that these basal zoosporic groups may contain substantial and largely unexplored diversity (Karpov et al. 2014, 2017; Letcher and Powell 2019), and comparable levels of hidden diversity have also been reported for the Ichthyosporea (Shabardina et al. 2024). It is possible that some of the unclassified OTU sequences represent deeply divergent lineages within these groups; however, additional research is needed to resolve their phylogenetic placement. For highly abundant taxa, direct metagenomic sequencing of DNA extracted from the water may provide a way to obtain genome‐level data and identify the organisms corresponding to these OTU sequences.

In relation to clearly identified zoosporic fungi, data from the present study indicate that ephemeral waterways within the Narrabri region act as hotspots for previously unknown chytrid and rozellid species. High abundances of zoosporic fungi have been reported in several habitats (Monchy et al. 2011; Panzer et al. 2015; Comeau et al. 2016; Rojas‐Jimenez et al. 2017), and their prevalence in this study extends such observations to include the Narrabri region. Continued efforts to isolate these lineages through baiting or related techniques may yield valuable insight into their ecological roles within this environment.

With respect to the Ascomycota, the absence of large numbers of ‘traditional’ aquatic hyphomycetes species may be related to the semi‐arid conditions in the region. As discussed previously, in this area many of the waterways or water bodies, apart from the Namoi River, are ephemeral. The timing of this pattern varies with weather conditions, but in most winters, smaller creeks and ponds tend to dry out or become fragmented. In many cases, this fragmentation results in discontinuous sections of surface flow, where isolated pools are separated by dry creek beds with shallow, subsurface flow occurring between these dry reaches through the sandy creek bed. The formation of these ‘sand barriers’ between pools in the dry season likely imparts significant selective pressure on the kinds of fungi within these communities (Mora‐Gómez et al. 2018; Gionchetta et al. 2020; Arias‐Real et al. 2022, 2023). These hyporheic sections of flow likely function as filters, preventing aquatic hyphomycetes from dispersing downstream—a mechanism demonstrated previously (Cornut et al. 2014). Further support for this supposition comes from the fact that the observed aquatic hyphomycete (OTU_4873; *Campylospora chaetocladia*) was only abundant in a major anabranch of the Namoi River—the largest and most reliably flowing watercourse in the region. Importantly, this study focused exclusively on the water column and did not sample from submerged plant detritus. It is possible that this detritus could support some aquatic hyphomycetes in the region, although confirming this would require a targeted future investigation. Regardless, the current study shows that conidia of these fungi were not present in the water column in notable quantities at either sampling time point.

Although this study was designed to compare freshwater fungal communities in summer (November) and winter (June), widespread flooding in November 2022 disrupted the seasonal contrast, conflating the effects of warmer temperatures with flood‐driven disturbances. Yet this flooding offers valuable insight, since the Narrabri region is inherently flood prone. The fungal data presented in this study demonstrates a significant difference (*p* < 0.0001) between the November and June collections, with November exhibiting both greater diversity and a markedly shifted community composition. Flooding is likely a contributing factor, as previous research has shown it can substantially impact microbial diversity. Caillon et al. (2021) showed that floodwaters rapidly inoculate aquatic systems with terrestrial and soil‐borne bacteria—likely including groundwater sources—mixing them with the indigenous microbes in the water. This process is likely exacerbated in semi‐arid regions as dust is generally high in these environments and heavy rainfall would mobilise this material from throughout the landscape and into the waterways.

At least some of the differences in this study between the two sampling points may have been related to seasonality. One example of this difference is a strain of *Laccaria miniata* (OTU_5520), which was detected in virtually all samples from the study site in June 2023, but was absent in November 2022. This species is an ectomycorrhizal basidiomycete and is almost certainly not aquatic. Notably, this is likely the first Australian record of the species (the OTU sequence is 99.6% identical to the reference sequence NR_198640), which was originally described from forest litter in southern China (Zhang et al. 2023). The frequent detection of this OTU in almost every water sample in June 2023 when flooding was absent suggests a mass fruiting event coincided with the collections in June 2023. The study area in question has an estimated size of above 2000 km^2^ suggesting that fruiting occurs at a similar time across a very large area. Mass fruiting events have been observed for at least some *Laccaria* species (Selosse et al. 2001), and presumably the same process is occurring here. Its relevance to the aquatic environment may lie in the large abundance of basidiospores entering the water, which likely provide a nutrient boon for organisms in the water column during this time of year.

Several nonfungal OTUs contributed significantly to differences in composition between seasons and deserve attention within the broader biogeographic context of this study. Although their identification was not a primary focus of this study, it offers new insights into the biogeography of these organisms that merit further discussion. Specifically, two freshwater sponge OTUs and a gastrotrich OTU were repeatedly detected. OTU_222 (*Heterorotula multidentata*; 100% ITS identity) was found in all November samples and most June samples but was most abundant in Yarrie Lake (W1). This species has been previously reported across parts of eastern Australia (Manconi et al. 2016; Belbin et al. 2021), although this is the first record of its presence in north‐western NSW, extending its distribution to the Narrabri region. The other freshwater sponge, OTU_245 ( *Anheteromeyenia argyrosperma* ; 99.68% ITS identity), was present in all the November samples but absent in June. To our knowledge, this is the first report of *Anheteromeyenia argyrosperma* in Australia. Its detection here may indicate either the introduction of this organism to the region or a broader, more cosmopolitan distribution than has been previously established. Previous reports indicate the taxon occurs mainly in North America. Surveys by De Santo and Fell (1996), Potts (1880a, 1880b), and Volkmer‐Ribeiro and Traveset (1987) recorded its presence in Connecticut and Pennsylvania, USA. The gastrotrich sequence (OTU_263) shows highest similarity to the red alga *Boldia erythrosiphon* (AF087109 and AF087113), while all subsequent matches correspond to invertebrate gastrotrich sequences assigned to the genus *Chaetonotus*. The closest identified species is *Chaetonotus gulosus* (OM421697), with 91% ITS identity. As gastrotrichs often feed on algae and may inhabit macroalgal surfaces (Ricci and Balsamo 2000), this sequence most likely represents a gastrotrich, and the initial *Boldia* alignments are best explained by contamination. Gastrotrichs are poorly studied in Australia (Balsamo et al. 2008), and this detection suggests that inland freshwater environments may harbour untapped gastrotrich diversity which is poorly represented in current reference databases.

## 5 Conclusions

This study provides the first insights into the fungal communities inhabiting water bodies in the semi‐arid Narrabri region of Australia. The data suggest that aquatic hyphomycetes are relatively scarce, while the water bodies of the region represent a hotspot for novel zoosporic fungal diversity. A substantial fraction of sequence reads could not be assigned to described taxa, highlighting a large component of unclassified fungal diversity (fungal dark matter) in these systems. Further research to determine the influence of hyporheic flow, perhaps through sampling water beneath the creek bed or in various niches, would likely provide additional understanding of the apparent absence of diverse aquatic hyphomycete lineages. Although this study initially aimed to explore the effects of seasonality on aquatic fungi, flooding during sampling conflated the summer collections with a flood event. Consequently, future research focusing on summer fungal diversity in the absence of flooding would be a valuable research direction for this region.

## Author Contributions

**Kim L. J. Porter:** conceptualization, writing – original draft, funding acquisition, investigation, visualization, formal analysis, data curation, validation, methodology, project administration. **Richard Schinteie:** methodology, visualization, writing – original draft, writing – review and editing. **Carla Mariani:** methodology, writing – review and editing. **Paul Greenfield:** methodology, writing – review and editing, writing – original draft, software, formal analysis, data curation, validation. **Se Gong:** methodology, writing – review and editing. **Stephen Sestak:** methodology, writing – review and editing. **Nai Tran‐Dinh:** methodology, validation, visualization, writing – original draft, writing – review and editing, investigation, conceptualization, formal analysis, data curation. **David J. Midgley:** conceptualization, investigation, funding acquisition, writing – original draft, writing – review and editing, visualization, validation, methodology, software, formal analysis, project administration, data curation, supervision.

## Funding

This work was supported by the CSIRO's Gas Industry Social and Environmental Research Alliance (GISERA) and CSIRO's Indigenous Graduate Programme.

## Conflicts of Interest

The authors declare no conflicts of interest.

## Supporting information


**Table S1:** Additional information on samples collected in this study.
**Table S2:** Complete OTU dataset, their GenBank information, and occurrences.
**Table S3:** Full chemistry profiles of water samples analysed in this study. All values are in mg L^−1^, unless specified otherwise.
**Table S4:** Comparison of ITS sequence identity across RefSeq, GenBank and UNITE for unclassified environmental OTUs.
**Table S5:** Top 20 OTUs driving community dissimilarity between June 2023 and November 2022 identified by SIMPER analysis.

## Data Availability

The data that supports the findings of this study are available in the Supporting Information of this article.
